# Massard Prairie Restoration and Soil Microbiome Succession

**Published:** 2020

**Authors:** J.M. Shaver, E.S. Bellis, C. Iwaki, J. Qualls, J. Randolph, J. Smith

**Affiliations:** 1 Department of Biology, University of Arkansas Fort Smith, Fort Smith, AR 72904; 2 Department of Computer Science, Arkansas State University, Jonesboro, AR 72467; 3 Arkansas Biosciences Institute, Arkansas State University, Jonesboro, AR 72467; 4 Department of Mathematics, University of Arkansas Fort Smith, Fort Smith, AR 72904; 5 Ben Geren Golf Course, Sebastian County, Fort Smith, AR 72903

## Abstract

We have initially sequenced soil microbial DNA from 4 restored and 3 virgin tallgrass prairie soil samples from Ben Geren Park and Massard Prairie (Fort Smith, AR), respectively. As expected, the soil microbiomes are distinct, with several lineages of nitrogen-fixing bacteria more common in virgin tallgrass prairie. However, we predict that as restoration of tallgrass prairie in Ben Geren Park progresses, the soil microbiome of restored prairie will more closely mirror those of the virgin prairie.

## Introduction

An ongoing project at Ben Geren Park (Fort Smith, AR) is the re-establishment of Massard Prairie, which once existed on the current site. Botanist Thomas Nuttall gave the first descriptive account of Massard Prairie in 1819, while visiting the newly established Fort Smith (Nuttall 1821). In Arkansas, tallgrass prairie once covered over 700,000 acres, but less than 0.5 percent remain today (Barone 2018). This makes tallgrass prairie one of the most rare and threatened ecosystems in the state. Prior to European settlement, North American prairie covered about 3.6 million-km^2^ (Mackelprang *et al.* 2018). Conversion to row crop agriculture and urban development has reduced North American prairies by 99.9% (Samson and Knopf 1994). This is of immediate concern, because prairie soils contain over 35% of soil carbon in the continental United States, making them some of the most productive and fertile in the world (Mackelprang *et al.* 2018).

The restoration areas in Ben Geren Park likely have altered microbial diversity and composition in the soil due to past cattle operations and maintenance practices involved in maintaining a golf course. The goal of this project is to establish a baseline for understanding the impacts of past land management and future prairie maintenance, development and restoration on soil microbial communities. In this preliminary study, 4 soil samples from developed prairie undergoing restoration that was initiated in 2016–2017, within Ben Geren Park, were compared to 3 virgin prairie samples obtained from Massard Prairie. The primary method we used for this study is 16S rRNA gene sequencing. LoopSeq synthetic long read sequencing, covering all 9 variable regions, facilitated species-level identification of soil bacteria discriminating virgin and restored tallgrass prairie soil samples.

## Materials and Methods

Four soil samples from restored prairie locations in Ben Geren Park and 3 soil samples from Massard Prairie were collected on February 10, 2019. Within each distinct sampling location, different soil types were taken into account when sample sites were identified, and soil temperatures and pH were also recorded, with GPS coordinates, as samples were collected (Table 1). All samples were taken at each of the sites with a 2.54-cm diameter soil corer to a depth of 10 cm. Each core sample was initially placed in a sterile plastic bucket and homogenized by hand. Buckets were sterilized by washing with soap and rinsed with isopropyl alcohol. After homogenization, a portion of the sample was placed in a sterile 50-ml plastic falcon tube and immediately placed on ice, and the bulk of the sample was placed in a thick paper bag for soil analysis. After all samples were collected, all samples on ice in 50-ml plastic falcon tubes were kept frozen in the lab at UA Fort Smith until DNA was extracted. A subsample of approximately 250 mg from each soil sample was used for DNA extraction and 16S rRNA gene sequencing.

DNA was extracted from 250 mg soil portions using the ZymoBIOMICS DNA Miniprep Kit (Cat # D4300) according to the manufacturer’s protocol (Zymo Research, Irvine, CA, United States). The V1-V9 region of the small subunit (SSU) rRNA gene was amplified using the LoopSeq^™^ 16S Long Read Kit according to the manufacturer’s protocol (Loop Genomics, San Jose, CA, United States). Sequencing of amplicons was performed at the Arkansas Childrens Research Institute (Little Rock, AR) using an Illumina sequencer following manufacturer’s instructions. For more information on 16S Long Read technology, see: https://www.loopgenomics.com/16s.

We used the linear discriminant analysis (LDA) effect size method (LEfSe) to identify particular microbial taxa associated with different soil types (Segata 2011). Using tabular data describing the relative abundance of bacterial ‘biomarkers’ in different samples, LEfSe identifies the taxa that most strongly differentiate two or more classes of samples. In this case, we used LEfSe to identify taxa that most strongly differentiated microbial communities from virgin and restored prairie soil samples.

We used the Galaxy implementation of LEfSe, with per-sample normalization to 1 M and default parameters, available at https://huttenhower.sph.harvard.edu/galaxy. Virgin and restored tallgrass prairie samples were also compared by Pearson’s Chi-square test using phylum counts and Principal Components Analysis (PCA) based on unscaled taxon abundances.

## Results

**Figure 1.**
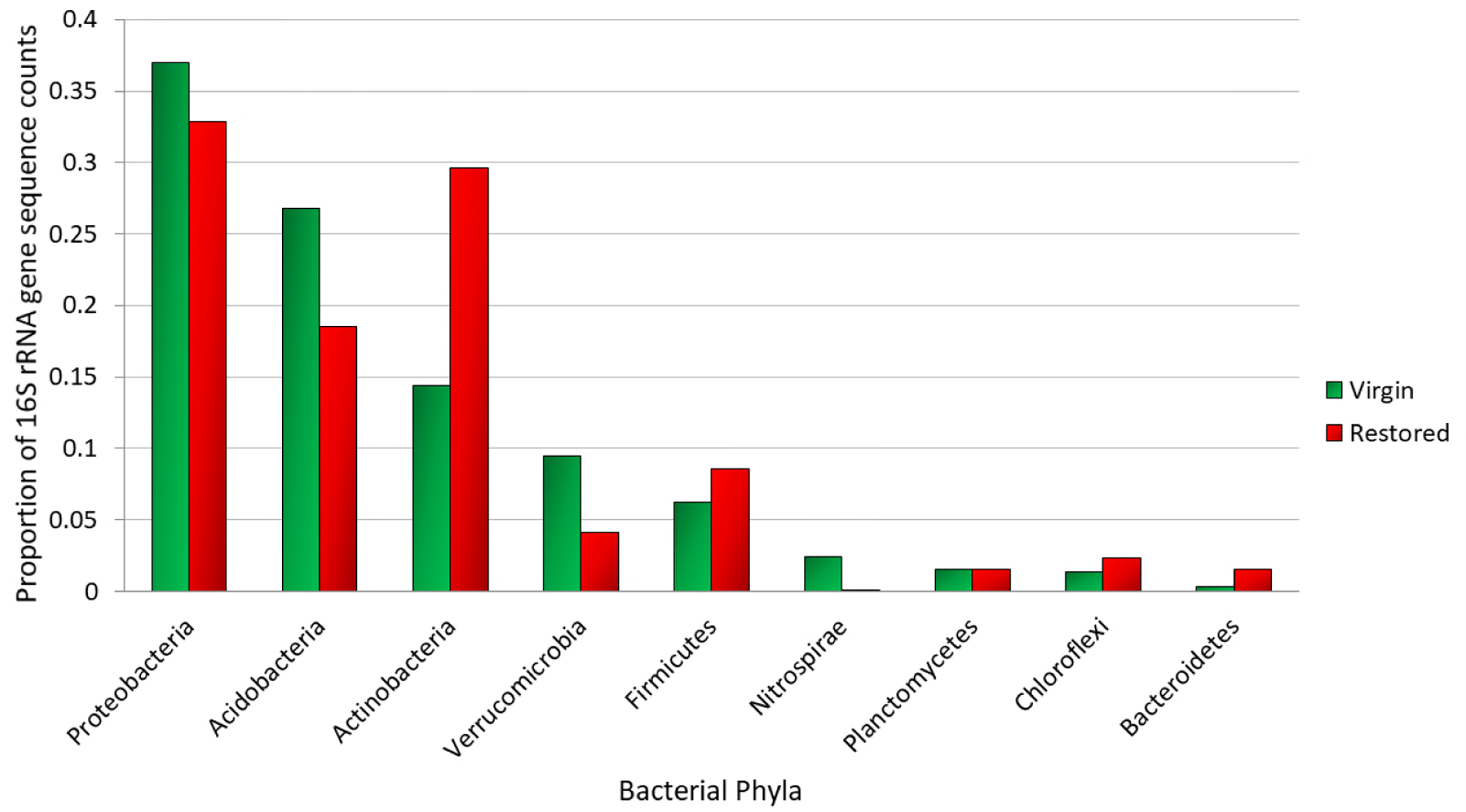
Proportion of 16S rRNA gene sequence counts for each bacterial phylum from the virgin and restored tallgrass prairie samples. The nine phyla included represent at least 97% of sequence counts. Samples were compared by Pearson’s Chi-square test using phylum counts. The P-value that resulted from the comparison of soil microbiome of virgin to restored tallgrass prairie soil samples was less than a significance level of 5%.

**Figure 2.**
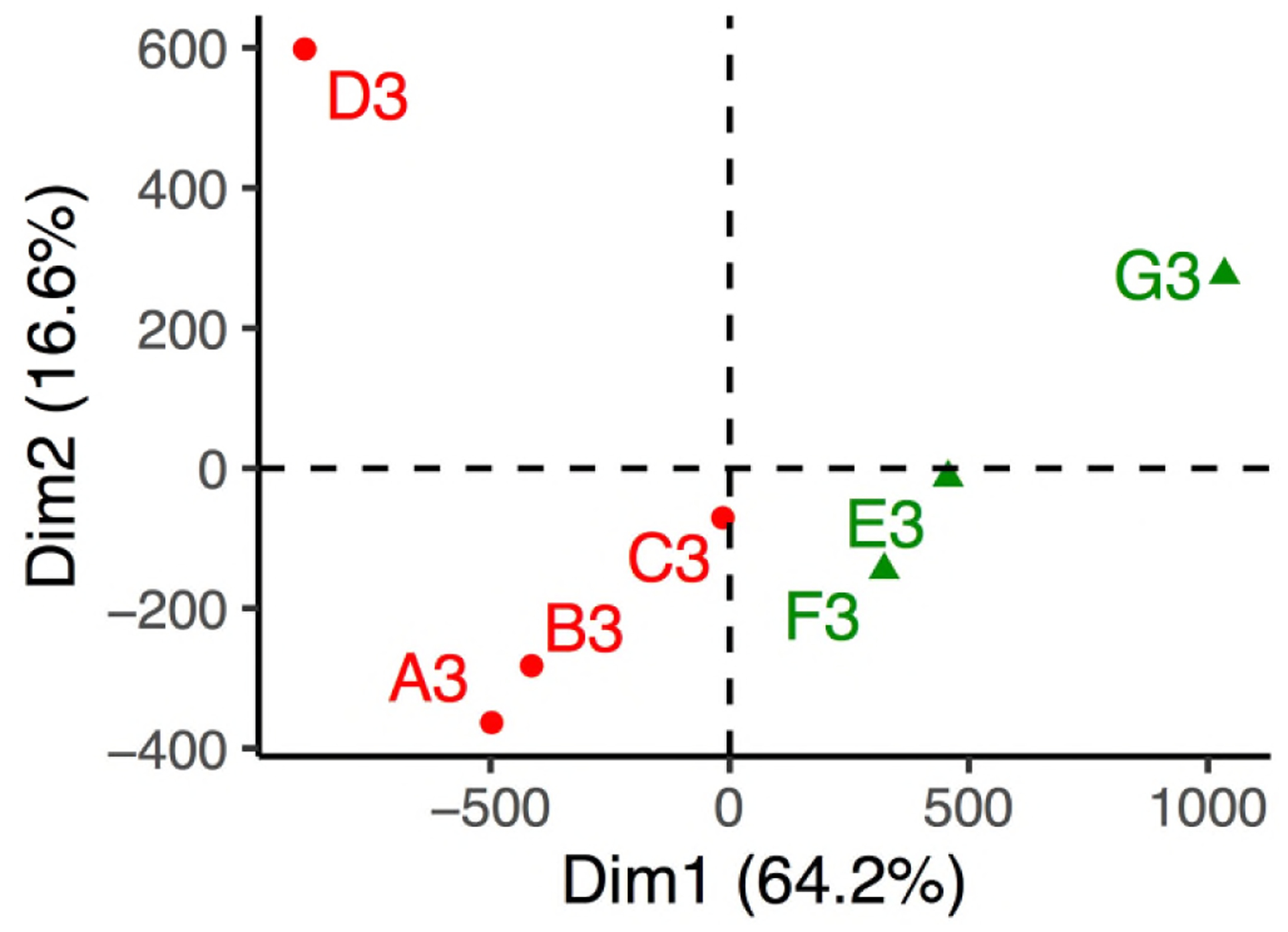
Principal Component Analysis (PCA) based on unscaled bacterial taxon abundance in virgin and restored prairie samples. Restored tallgrass prairie soil samples shown as red circles. Virgin tallgrass prairie soil samples shown as green triangles.

The proportion of 16S rRNA sequence counts for each bacterial phylum from the virgin and restored tallgrass prairie samples were compared (Fig. 1). Nine phyla compared represent at least 97% of the sequence counts. Virgin and restored tallgrass prairie samples were compared by Pearson’s Chi-square test using phylum counts. The P-value (0.000) that resulted from the comparison of soil microbiome of virgin to restored tallgrass prairie soil samples was less than a significance level of 5% (8 degrees of freedom). Virgin and restored samples also clustered separately along the first principal component dimension, which explained 64.2% of total variation in taxon abundances among samples (Fig. 2).

**Figure 3.**
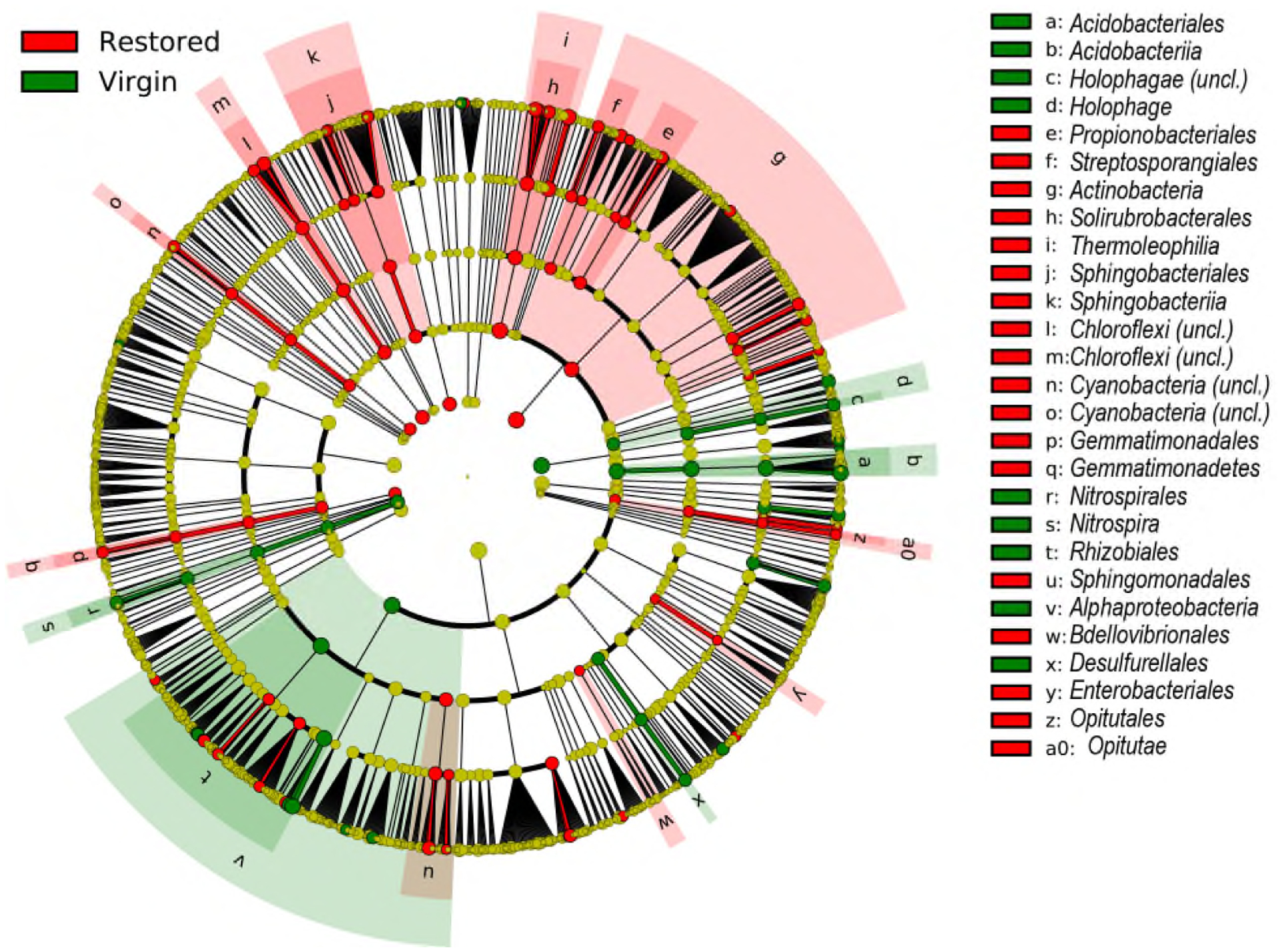
Cladogram representing LEfSe (Linear Discriminant Analysis Effect Size) results featuring bacterial taxa most likely to explain differences between Ben Geren Park and Massard Prairie soil samples. Each ‘ring’ in the figure represents a different hierarchical level of classification, with nodes in the innermost ring representing different bacterial phyla. Nodes are colored if they represent a lineage significantly more abundant in restored (red) or virgin (green) tallgrass prairie samples at each level of taxonomic classification. Bacterial classes (second ring) and orders (third ring) that are significantly differentiated between restored and virgin samples are shaded on the cladogram and given an abbreviated label (see legend).

Eighty-six lineages of bacteria were significantly more common in virgin prairie samples compared to the restored prairie samples (Fig. 3). Four classes overall were more abundant in virgin prairie: *Alphaproteobacteria*, *Acidobacteriia*, *Holophagae*, and *Nitrospira*. The higher abundance of *Alphaproteobacteria* in virgin prairie soils was driven by several lineages of *Rhizobiales*, including members of the *Xanthobacteraceae*, *Methylobacteriaceae*, *Hyphomicrobiaceae*, and *Bradyrhizobiaceae*. In contrast, 123 lineages were more common in restored prairie samples. Seven classes were more abundant in restored prairie samples, including *Actinobacteria* (particularly the families *Propionibacteriales* and *Streptosporangiales*), *Gemmatimonadetes*, *Opitutae*, *Sphingobacteriia*, *Thermoleophilia*, and unclassified members of *Cyanobacteria* and *Chloroflexi*.

## Discussion

Even with a small sample size, we identified several bacterial taxa that have previously been suggested as bioindicators of healthy prairie soils. In our study, one of the most strongly differentiating families was the *Xanthobacteraceae* within *Rhizobiales*; several particular lineages within three other families of *Rhizobiales* were also more significantly abundant in virgin prairie. Multiple families of *Rhizobiales* were more commonly associated with prairie soils compared to soils under corn cultivation (Mackelprang *et al.* 2018). Many rhizosphere-associated taxa, including most lineages of symbiotic nitrogen-fixing rhizobia, are classified within families of the *Rhizobiales*.

Within the *Verrucomicrobia*, the *Spartobacteria* are a taxon of interest for tallgrass prairie restoration efforts, given their likely importance for carbon dynamics (Fierer *et al.* 2013) and abundance in older, 10+ year-old restorations and virgin prairie (Barber *et al.* 2017). Of the *Spartobacteria* detected in our study, members of the *Xiphinematobacteraceae* and an unclassified family within the *Chthoniobacterales* were more abundant in virgin soils compared to restored prairie soils. The *Xiphinematobacteraceae* lineages detected were all most similar to ‘*Candidatus Xiphinematobacter*’, endosymbionts of soil nematodes (Brown *et al.* 2015). Another verrucomicrobial taxon, the *Opitutae*, were slightly more abundant in restorations in our study compared to virgin prairie, consistent with their higher abundance in early restorations and decrease over time in older restorations (Barber *et al.* 2017).

Surprisingly, we found that *Nitrospirales* were more common in virgin soils than restored prairie soils. *Nitrospirales*, involved in nitrification, were more abundant under corn cultivation than in virgin prairie in a previous study (Mackelprang *et al.* 2018).

For future study, we will sequence bacterial and fungal DNA from 12 new soil samples taken in March 2020 from locations in Ben Geren Park and Massard Prairie, and sequence fungal DNA from 12 soil samples collected from the same locations in January 2019. LoopSeq synthetic long read sequencing (generating ~2.5 kb contigs covering the 18S-ITS1-ITS2 region) will facilitate species level identification of soil fungi present in these 24 samples We will explore the use of multilayer networks for analysis of co-occurrence data, wherein each layer represents a different sampling date, and inter-layer edges represent temporal change in abundance of a particular taxon. We hypothesize higher fungal taxonomic diversity in the virgin soils and an increase in fungal diversity over time under restorations.

## Figures and Tables

**Table 1.** Soil sample collection information, including GPS coordinates location, pH, temperature, and soil description. Restoration efforts for sample areas began in 2016 (Sample 1) and 2017 (Samples 2–4).

Sample	GPS	Prairie Location	pH	Temp (°C)	Soil Description
1 (A3)	N 35.314230 W 94.362009	Restored	5.29	5.5	Wt - Wrightsville Messer complex
2 (B3)	N 35.313273 W 94.361.789	Restored	5.00	5.0	Wt - Wrightsville Messer complex
3 (C3)	N 35.315214 W 94.358842	Restored	4.88	4.4	Wt - Wrightsville Messer complex
4 (D3)	N 35.317022 W 94.359211	Restored	4.96	4.9	WsA - Wrightsville complex
5 (E3)	N 35.294147 W 94.380950	Virgin	5.59	5.2	LeB - Leadvale Silt Loam
6 (F3)	N 35.298083 W 94.381193	Virgin	5.28	5.6	WsA - Wrightsville complex
7 (G3)	N 35.297956 W 94.384681	Virgin	5.13	6.0	MID - Montevallo gravelly loam
